# Caffeine–*N*-phthaloyl-β-alanine (1/1)

**DOI:** 10.1107/S1600536812022696

**Published:** 2012-05-26

**Authors:** Moazzam H. Bhatti, Uzma Yunus, Syed Raza Shah, Ulrich Flörke

**Affiliations:** a Department of Chemistry, Allama Iqbal Open University, Islamabad, Pakistan; b Anorganische und Analytische Chemie, Fakultät für Naturwissenschaften, Universität Paderborn, Warburgerstrasse 100, D-33098 Paderborn, Germany

## Abstract

The title co-crystal [systematic name: 3-(1,3-dioxoisoindolin-2-yl)propanoic acid–1,3,7-trimethyl-1*H*-purine-2,6(3*H*,7*H*)-dione (1/1)], C_8_H_10_N_4_O_2_·C_11_H_9_NO_4_, is the combination of 1:1 adduct of *N*-phthaloyl-β-alanine with caffeine. The phthalimide and purine rings in the *N*-phthaloyl-β-alanine and caffeine mol­ecules are essentially planar, with r.m.s. deviations of the fitted atoms of 0.0078 and 0.0118 Å, respectively. In the crystal, the two mol­ecules are linked *via* an O—H⋯N hydrogen bond involving the intact carb­oxy­lic acid (COOH) group. The crystal structure is consolidated by C—H⋯O inter­actions. The H atoms of a methyl group of the caffeine mol­ecule are disordered over two sets of sites of equal occupancy.

## Related literature

For related structures, see: Bhatti *et al.* (2011 ▶); Feeder & Jones (1996 ▶). 
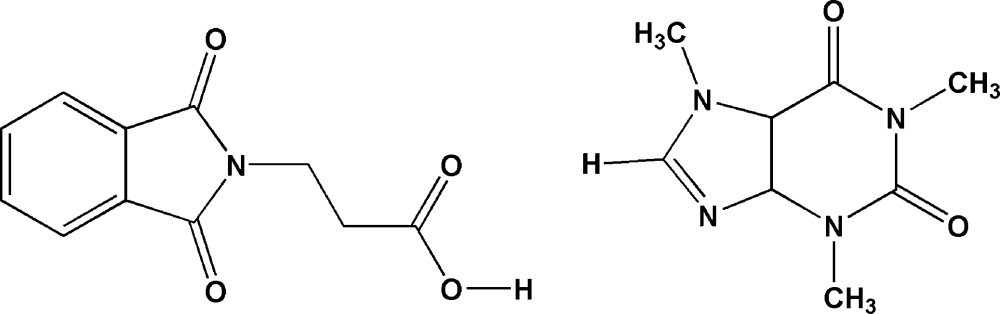


## Experimental

### 

#### Crystal data

C_8_H_10_N_4_O_2_·C_11_H_9_NO_4_
*M* *_r_* = 413.39
Triclinic, 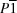

*a* = 8.3411 (17) Å
*b* = 9.0638 (18) Å
*c* = 13.162 (3) Å
α = 77.105 (4)°
β = 82.394 (4)°
γ = 72.865 (4)°
*V* = 924.6 (3) Å^3^
*Z* = 2
Mo *K*α radiation
μ = 0.11 mm^−1^
*T* = 130 K
0.42 × 0.40 × 0.35 mm

#### Data collection

Bruker SMART APEX diffractometer
Absorption correction: multi-scan (*SADABS*; Sheldrick, 2004 ▶) *T*_min_ = 0.954, *T*_max_ = 0.962
8826 measured reflections
4378 independent reflections
3752 reflections with *I* > 2σ(*I*)
*R*_int_ = 0.022

#### Refinement

*R*[*F* ^2^ > 2σ(*F* ^2^)] = 0.039
*wR*(*F* ^2^) = 0.106
*S* = 1.03
4378 reflections
277 parameters
H-atom parameters constrained
Δρ_max_ = 0.26 e Å^−3^
Δρ_min_ = −0.26 e Å^−3^

### 

Data collection: *SMART* (Bruker, 2002 ▶); cell refinement: *SAINT* (Bruker, 2002 ▶); data reduction: *SAINT*; program(s) used to solve structure: *SHELXS97* (Sheldrick, 2008 ▶); program(s) used to refine structure: *SHELXL97* (Sheldrick, 2008 ▶); molecular graphics: *SHELXTL* (Sheldrick, 2008 ▶); software used to prepare material for publication: *SHELXTL* and local programs.

## Supplementary Material

Crystal structure: contains datablock(s) I, global. DOI: 10.1107/S1600536812022696/pv2544sup1.cif

Structure factors: contains datablock(s) I. DOI: 10.1107/S1600536812022696/pv2544Isup2.hkl

Supplementary material file. DOI: 10.1107/S1600536812022696/pv2544Isup3.cml

Additional supplementary materials:  crystallographic information; 3D view; checkCIF report

## Figures and Tables

**Table 1:** Hydrogen-bond geometry (Å, °)

*D*—H⋯*A*	*D*—H	H⋯*A*	*D*⋯*A*	*D*—H⋯*A*
O4—H4⋯N3^i^	0.84	1.83	2.6672 (13)	175
C3—H3*A*⋯O5^ii^	0.95	2.26	3.1447 (16)	155
C6—H6*A*⋯O3^iii^	0.95	2.31	3.2283 (16)	162
C20—H20*B*⋯O6^iv^	0.98	2.35	3.2559 (16)	154

Symmetry codes: (i) 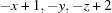; (ii) 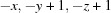; (iii) 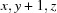; (iv) 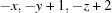.

**Fig. 1.**
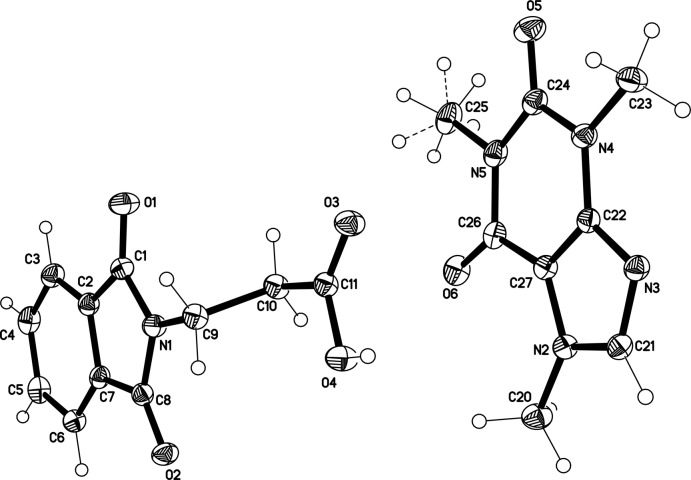
The molecular structure of the title adduct with the atom numbering scheme. Displacement ellipsoids are drawn at the 30% probability level. H atoms are presented as small spheres of arbitrary radius.

**Fig. 2.**
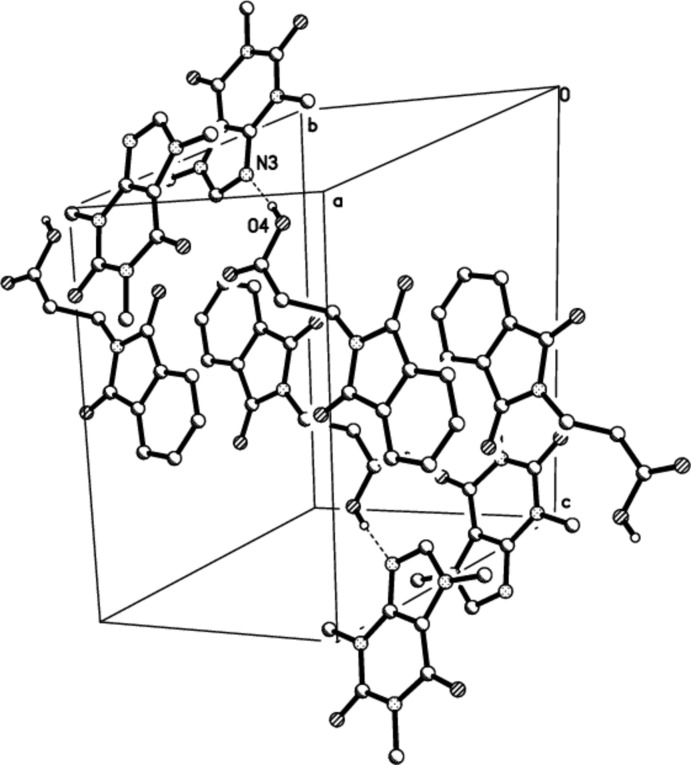
A view of the O—H···N hydrogen bonds (dotted lines) in the crystal structure of the title compound. H atoms non-participating in hydrogen- bonding were omitted for clarity.

## supplementary crystallographic information

### Comment

Previously we have reported the synthesis and crystal structure of a 1:1 adduct of *N*-phthaloylglycine with caffeine (Bhatti *et al.*, 2011). Now we have synthesized a 1:1 adduct of *N*-phthaloyl-β-alanine with caffeine and determined its crystal structure which is reported in this article.

The asymmetric unit of the title adduct is presented in Figure 1. The phthalimide and purine rings in the *N*-phthaloyl-β-alanine and caffeine molecules are essentailly planar with rms deviations of fitted atoms 0.0078 and 0.0118 Å, respectively; the dihedral angle between the mean-planes of these rings is 5.59 (5)°. The dihedral angle between phthlimide and propanoic acid is 6.5 (1)° slightly less than reported value of *N*-phthaloyl-β-alanine (Feeder & Jones, 1996). The carbon oxygen distance in the carboxylic acid group (COOH) show typical double and single bond values [C11—O3 = 1.2066 (15) Å and C11—O4 = 1.3312 (14) Å, respectively)], indicating intact protonation of carboxylic acid group. This is further strengthened by intermolecular O4—H4···N3 hydrogen bonding which link the two molecules (Fig. 2). The crystal structure is further consolidated by C—H···O type intermolecular interactions (Table 1).

### Experimental

A mixture of *N*-phthaloyl-β-alanine (0.01 mol) and caffeine (0.01 mol) was heated in water (100 ml) for 2 h. The hot solution was filtered and the filtrate was set aside for one week. Colourless needle like crystals were obtained suitable for X-ray analysis.

### Refinement

Although all hydrogen atoms were clearly identified in difference syntheses, they were positioned geometrically and refined using a riding model, with O—H = 0.84 Å and C—H = 0.95, 0.98 and 0.99 Å, for aryl, methyl and methylene H-atoms, respectively. The *U*_iso_(H) were allowed at 1.5*U*_eq_(O/C methyl) or 1.2*U*_eq_(C non-methyl). The hydrogen atoms of the C25 methyl group of caffeine molecule are disordered over two positions with site occupation of 0.5 each.

### Crystal data

**Table:**

C_8_H_10_N_4_O_2_·C_11_H_9_NO_4_	*Z* = 2
*M**_r_* = 413.39	*F*(000) = 432
Triclinic, *P*1	*D*_x_ = 1.485 Mg m^−^^3^
Hall symbol: -P 1	Mo *K*α radiation, λ = 0.71073 Å
*a* = 8.3411 (17) Å	Cell parameters from 3614 reflections
*b* = 9.0638 (18) Å	θ = 2.6–28.2°
*c* = 13.162 (3) Å	µ = 0.11 mm^−^^1^
α = 77.105 (4)°	*T* = 130 K
β = 82.394 (4)°	Block, colourless
γ = 72.865 (4)°	0.42 × 0.40 × 0.35 mm
*V* = 924.6 (3) Å^3^	

### Data collection

**Table:**

Bruker SMART APEX diffractometer	4378 independent reflections
Radiation source: sealed tube	3752 reflections with *I* > 2σ(*I*)
Graphite monochromator	*R*_int_ = 0.022
φ and ω scans	θ_max_ = 27.9°, θ_min_ = 1.6°
Absorption correction: multi-scan (*SADABS*; Sheldrick, 2004)	*h* = −10→10
*T*_min_ = 0.954, *T*_max_ = 0.962	*k* = −11→11
8826 measured reflections	*l* = −17→17

### Refinement

**Table:**

Refinement on *F*^2^	Secondary atom site location: difference Fourier map
Least-squares matrix: full	Hydrogen site location: difference Fourier map
*R*[*F*^2^ > 2σ(*F*^2^)] = 0.039	H-atom parameters constrained
*wR*(*F*^2^) = 0.106	*w* = 1/[σ^2^(*F*_o_^2^) + (0.0581*P*)^2^ + 0.1919*P*] where *P* = (*F*_o_^2^ + 2*F*_c_^2^)/3
*S* = 1.03	(Δ/σ)_max_ < 0.001
4378 reflections	Δρ_max_ = 0.26 e Å^−^^3^
277 parameters	Δρ_min_ = −0.26 e Å^−^^3^
0 restraints	Extinction correction: *SHELXL97* (Sheldrick, 2008), Fc^*^=kFc[1+0.001xFc^2^λ^3^/sin(2θ)]^-1/4^
Primary atom site location: structure-invariant direct methods	Extinction coefficient: 0.0023 (15)

### Special details

**Table:**

Geometry. All e.s.d.'s (except the e.s.d. in the dihedral angle between two l.s. planes) are estimated using the full covariance matrix. The cell e.s.d.'s are taken into account individually in the estimation of e.s.d.'s in distances, angles and torsion angles; correlations between e.s.d.'s in cell parameters are only used when they are defined by crystal symmetry. An approximate (isotropic) treatment of cell e.s.d.'s is used for estimating e.s.d.'s involving l.s. planes.
Refinement. Refinement of *F*^2^ against ALL reflections. The weighted *R*-factor *wR* and goodness of fit *S* are based on *F*^2^, conventional *R*-factors *R* are based on *F*, with *F* set to zero for negative *F*^2^. The threshold expression of *F*^2^ > σ(*F*^2^) is used only for calculating *R*-factors(gt) *etc*. and is not relevant to the choice of reflections for refinement. *R*-factors based on *F*^2^ are statistically about twice as large as those based on *F*, and *R*- factors based on ALL data will be even larger.

### Fractional atomic coordinates and isotropic or equivalent isotropic displacement parameters (Å^2^)

**Table:**

	*x*	*y*	*z*	*U*_iso_*/*U*_eq_	Occ. (<1)
O1	0.27140 (12)	0.71481 (11)	0.44923 (7)	0.0325 (2)	
O2	0.44166 (11)	0.89747 (10)	0.70215 (7)	0.0269 (2)	
O3	0.39875 (12)	0.24964 (10)	0.74716 (7)	0.0289 (2)	
O4	0.49142 (12)	0.38406 (10)	0.83878 (7)	0.0289 (2)	
H4	0.5451	0.2935	0.8673	0.043*	
N1	0.37128 (13)	0.76999 (12)	0.58804 (8)	0.0228 (2)	
C1	0.28998 (15)	0.80945 (15)	0.49543 (9)	0.0236 (2)	
C2	0.23764 (14)	0.98470 (14)	0.46992 (9)	0.0223 (2)	
C3	0.15355 (15)	1.08616 (16)	0.38653 (10)	0.0273 (3)	
H3A	0.1171	1.0480	0.3346	0.033*	
C4	0.12468 (15)	1.24709 (16)	0.38213 (10)	0.0290 (3)	
H4A	0.0670	1.3201	0.3261	0.035*	
C5	0.17853 (15)	1.30301 (15)	0.45800 (10)	0.0279 (3)	
H5A	0.1573	1.4133	0.4526	0.034*	
C6	0.26336 (15)	1.19969 (15)	0.54206 (10)	0.0247 (3)	
H6A	0.3006	1.2372	0.5941	0.030*	
C7	0.29048 (14)	1.04076 (14)	0.54603 (9)	0.0215 (2)	
C8	0.37667 (14)	0.90240 (14)	0.62382 (9)	0.0214 (2)	
C9	0.44832 (15)	0.61010 (14)	0.63967 (10)	0.0236 (2)	
H9A	0.5306	0.6112	0.6870	0.028*	
H9B	0.5099	0.5479	0.5866	0.028*	
C10	0.31773 (15)	0.53188 (14)	0.70237 (10)	0.0239 (3)	
H10A	0.2477	0.5993	0.7505	0.029*	
H10B	0.2431	0.5189	0.6544	0.029*	
C11	0.40448 (14)	0.37333 (14)	0.76419 (9)	0.0219 (2)	
O5	0.05201 (12)	−0.05872 (12)	0.78890 (7)	0.0323 (2)	
O6	0.06764 (11)	0.39207 (10)	0.88703 (7)	0.0292 (2)	
N2	0.28028 (12)	0.17054 (11)	1.06458 (8)	0.0210 (2)	
N3	0.34827 (12)	−0.08987 (12)	1.07618 (8)	0.0211 (2)	
N4	0.19842 (12)	−0.08902 (12)	0.92895 (8)	0.0225 (2)	
N5	0.05480 (12)	0.16501 (12)	0.84150 (8)	0.0246 (2)	
C20	0.27113 (17)	0.32244 (15)	1.08893 (11)	0.0292 (3)	
H20A	0.3295	0.3056	1.1522	0.044*	
H20B	0.1530	0.3804	1.1004	0.044*	
H20C	0.3247	0.3833	1.0305	0.044*	
C21	0.36256 (14)	0.02847 (13)	1.11659 (9)	0.0214 (2)	
H21A	0.4241	0.0136	1.1756	0.026*	
C22	0.25050 (14)	−0.01704 (14)	0.99472 (9)	0.0196 (2)	
C23	0.25004 (16)	−0.26028 (15)	0.93844 (10)	0.0273 (3)	
H23A	0.3469	−0.2903	0.8890	0.041*	
H23B	0.1567	−0.2948	0.9229	0.041*	
H23C	0.2811	−0.3105	1.0098	0.041*	
C24	0.09971 (14)	0.00176 (15)	0.84884 (9)	0.0239 (3)	
C25	−0.04962 (17)	0.26056 (18)	0.75566 (10)	0.0330 (3)	
H25A	−0.0890	0.3702	0.7647	0.050*	0.50
H25B	−0.1466	0.2202	0.7562	0.050*	0.50
H25C	0.0172	0.2549	0.6888	0.050*	0.50
H25D	−0.0567	0.1933	0.7085	0.050*	0.50
H25E	0.0010	0.3434	0.7170	0.050*	0.50
H25F	−0.1628	0.3086	0.7844	0.050*	0.50
C26	0.10740 (14)	0.24778 (14)	0.90378 (9)	0.0226 (2)	
C27	0.20625 (14)	0.14314 (14)	0.98483 (9)	0.0206 (2)	

### Atomic displacement parameters (Å^2^)

**Table:**

	*U*^11^	*U*^22^	*U*^33^	*U*^12^	*U*^13^	*U*^23^
O1	0.0409 (5)	0.0297 (5)	0.0309 (5)	−0.0097 (4)	−0.0096 (4)	−0.0099 (4)
O2	0.0321 (5)	0.0264 (5)	0.0230 (4)	−0.0075 (4)	−0.0101 (4)	−0.0023 (4)
O3	0.0373 (5)	0.0201 (4)	0.0304 (5)	−0.0067 (4)	−0.0098 (4)	−0.0040 (4)
O4	0.0360 (5)	0.0177 (4)	0.0330 (5)	−0.0036 (4)	−0.0161 (4)	−0.0018 (4)
N1	0.0269 (5)	0.0189 (5)	0.0222 (5)	−0.0059 (4)	−0.0063 (4)	−0.0012 (4)
C1	0.0247 (6)	0.0257 (6)	0.0212 (6)	−0.0077 (5)	−0.0033 (4)	−0.0044 (5)
C2	0.0211 (5)	0.0241 (6)	0.0208 (6)	−0.0060 (4)	−0.0021 (4)	−0.0022 (5)
C3	0.0243 (6)	0.0344 (7)	0.0223 (6)	−0.0072 (5)	−0.0055 (5)	−0.0027 (5)
C4	0.0223 (6)	0.0315 (7)	0.0254 (6)	−0.0010 (5)	−0.0048 (5)	0.0036 (5)
C5	0.0253 (6)	0.0218 (6)	0.0309 (7)	−0.0016 (5)	−0.0005 (5)	−0.0009 (5)
C6	0.0256 (6)	0.0234 (6)	0.0247 (6)	−0.0059 (5)	−0.0026 (5)	−0.0047 (5)
C7	0.0208 (5)	0.0229 (6)	0.0189 (5)	−0.0050 (4)	−0.0022 (4)	−0.0011 (4)
C8	0.0208 (5)	0.0220 (6)	0.0210 (6)	−0.0063 (4)	−0.0023 (4)	−0.0024 (4)
C9	0.0241 (6)	0.0187 (6)	0.0259 (6)	−0.0033 (4)	−0.0048 (5)	−0.0020 (5)
C10	0.0243 (6)	0.0193 (6)	0.0270 (6)	−0.0047 (4)	−0.0064 (5)	−0.0015 (5)
C11	0.0222 (5)	0.0199 (6)	0.0230 (6)	−0.0051 (4)	−0.0032 (4)	−0.0028 (4)
O5	0.0318 (5)	0.0408 (6)	0.0277 (5)	−0.0105 (4)	−0.0103 (4)	−0.0083 (4)
O6	0.0284 (4)	0.0223 (4)	0.0298 (5)	0.0016 (3)	−0.0047 (4)	−0.0002 (4)
N2	0.0210 (5)	0.0192 (5)	0.0217 (5)	−0.0028 (4)	−0.0030 (4)	−0.0042 (4)
N3	0.0215 (5)	0.0206 (5)	0.0202 (5)	−0.0040 (4)	−0.0048 (4)	−0.0022 (4)
N4	0.0241 (5)	0.0229 (5)	0.0210 (5)	−0.0062 (4)	−0.0053 (4)	−0.0034 (4)
N5	0.0220 (5)	0.0288 (6)	0.0200 (5)	−0.0049 (4)	−0.0064 (4)	0.0009 (4)
C20	0.0344 (7)	0.0211 (6)	0.0321 (7)	−0.0029 (5)	−0.0060 (5)	−0.0094 (5)
C21	0.0206 (5)	0.0211 (6)	0.0208 (6)	−0.0031 (4)	−0.0040 (4)	−0.0028 (4)
C22	0.0179 (5)	0.0216 (6)	0.0185 (5)	−0.0052 (4)	−0.0016 (4)	−0.0024 (4)
C23	0.0314 (6)	0.0225 (6)	0.0302 (6)	−0.0069 (5)	−0.0068 (5)	−0.0075 (5)
C24	0.0202 (5)	0.0306 (6)	0.0205 (6)	−0.0067 (5)	−0.0032 (4)	−0.0036 (5)
C25	0.0287 (6)	0.0403 (8)	0.0237 (6)	−0.0041 (6)	−0.0105 (5)	0.0046 (6)
C26	0.0180 (5)	0.0243 (6)	0.0213 (6)	−0.0021 (4)	−0.0004 (4)	−0.0013 (5)
C27	0.0190 (5)	0.0208 (6)	0.0208 (6)	−0.0036 (4)	−0.0025 (4)	−0.0032 (4)

### Geometric parameters (Å, º)

**Table:**

O1—C1	1.2106 (15)	O6—C26	1.2265 (15)
O2—C8	1.2132 (14)	N2—C21	1.3446 (15)
O3—C11	1.2066 (15)	N2—C27	1.3867 (15)
O4—C11	1.3312 (14)	N2—C20	1.4604 (16)
O4—H4	0.8400	N3—C21	1.3395 (15)
N1—C8	1.3979 (16)	N3—C22	1.3597 (14)
N1—C1	1.3982 (15)	N4—C22	1.3719 (15)
N1—C9	1.4499 (15)	N4—C24	1.3820 (15)
C1—C2	1.4895 (17)	N4—C23	1.4644 (16)
C2—C3	1.3841 (16)	N5—C24	1.3994 (17)
C2—C7	1.3899 (17)	N5—C26	1.4110 (16)
C3—C4	1.3957 (19)	N5—C25	1.4712 (15)
C3—H3A	0.9500	C20—H20A	0.9800
C4—C5	1.3910 (19)	C20—H20B	0.9800
C4—H4A	0.9500	C20—H20C	0.9800
C5—C6	1.3986 (17)	C21—H21A	0.9500
C5—H5A	0.9500	C22—C27	1.3689 (16)
C6—C7	1.3807 (17)	C23—H23A	0.9800
C6—H6A	0.9500	C23—H23B	0.9800
C7—C8	1.4927 (16)	C23—H23C	0.9800
C9—C10	1.5252 (17)	C25—H25A	0.9800
C9—H9A	0.9900	C25—H25B	0.9800
C9—H9B	0.9900	C25—H25C	0.9800
C10—C11	1.5073 (16)	C25—H25D	0.9800
C10—H10A	0.9900	C25—H25E	0.9800
C10—H10B	0.9900	C25—H25F	0.9800
O5—C24	1.2190 (15)	C26—C27	1.4275 (16)
			
C11—O4—H4	109.5	C21—N3—C22	104.19 (10)
C8—N1—C1	112.53 (10)	C22—N4—C24	119.59 (10)
C8—N1—C9	123.16 (10)	C22—N4—C23	121.80 (10)
C1—N1—C9	124.26 (10)	C24—N4—C23	118.57 (10)
O1—C1—N1	124.51 (12)	C24—N5—C26	126.81 (10)
O1—C1—C2	130.13 (11)	C24—N5—C25	116.45 (10)
N1—C1—C2	105.36 (10)	C26—N5—C25	116.62 (11)
C3—C2—C7	121.42 (12)	N2—C20—H20A	109.5
C3—C2—C1	130.01 (11)	N2—C20—H20B	109.5
C7—C2—C1	108.56 (10)	H20A—C20—H20B	109.5
C2—C3—C4	117.05 (12)	N2—C20—H20C	109.5
C2—C3—H3A	121.5	H20A—C20—H20C	109.5
C4—C3—H3A	121.5	H20B—C20—H20C	109.5
C5—C4—C3	121.47 (11)	N3—C21—N2	112.65 (10)
C5—C4—H4A	119.3	N3—C21—H21A	123.7
C3—C4—H4A	119.3	N2—C21—H21A	123.7
C4—C5—C6	121.12 (12)	N3—C22—C27	111.37 (10)
C4—C5—H5A	119.4	N3—C22—N4	126.43 (11)
C6—C5—H5A	119.4	C27—C22—N4	122.20 (10)
C7—C6—C5	116.97 (11)	N4—C23—H23A	109.5
C7—C6—H6A	121.5	N4—C23—H23B	109.5
C5—C6—H6A	121.5	H23A—C23—H23B	109.5
C6—C7—C2	121.96 (11)	N4—C23—H23C	109.5
C6—C7—C8	130.04 (11)	H23A—C23—H23C	109.5
C2—C7—C8	107.99 (10)	H23B—C23—H23C	109.5
O2—C8—N1	124.43 (11)	O5—C24—N4	121.12 (12)
O2—C8—C7	130.01 (11)	O5—C24—N5	121.97 (11)
N1—C8—C7	105.56 (10)	N4—C24—N5	116.90 (10)
N1—C9—C10	111.68 (10)	N5—C25—H25A	109.5
N1—C9—H9A	109.3	N5—C25—H25B	109.5
C10—C9—H9A	109.3	H25A—C25—H25B	109.5
N1—C9—H9B	109.3	N5—C25—H25C	109.5
C10—C9—H9B	109.3	H25A—C25—H25C	109.5
H9A—C9—H9B	107.9	H25B—C25—H25C	109.5
C11—C10—C9	109.85 (10)	N5—C25—H25D	109.5
C11—C10—H10A	109.7	N5—C25—H25E	109.5
C9—C10—H10A	109.7	H25D—C25—H25E	109.5
C11—C10—H10B	109.7	N5—C25—H25F	109.5
C9—C10—H10B	109.7	H25D—C25—H25F	109.5
H10A—C10—H10B	108.2	H25E—C25—H25F	109.5
O3—C11—O4	123.34 (11)	O6—C26—N5	121.50 (11)
O3—C11—C10	124.00 (11)	O6—C26—C27	126.91 (12)
O4—C11—C10	112.64 (10)	N5—C26—C27	111.59 (10)
C21—N2—C27	106.39 (10)	C22—C27—N2	105.40 (10)
C21—N2—C20	126.14 (10)	C22—C27—C26	122.76 (11)
C27—N2—C20	127.47 (10)	N2—C27—C26	131.82 (11)
			
C8—N1—C1—O1	179.26 (12)	C27—N2—C21—N3	0.32 (13)
C9—N1—C1—O1	1.75 (19)	C20—N2—C21—N3	179.91 (11)
C8—N1—C1—C2	−0.27 (13)	C21—N3—C22—C27	0.33 (13)
C9—N1—C1—C2	−177.79 (10)	C21—N3—C22—N4	−179.28 (11)
O1—C1—C2—C3	0.1 (2)	C24—N4—C22—N3	−179.58 (11)
N1—C1—C2—C3	179.57 (12)	C23—N4—C22—N3	−2.06 (18)
O1—C1—C2—C7	−178.84 (13)	C24—N4—C22—C27	0.86 (17)
N1—C1—C2—C7	0.66 (13)	C23—N4—C22—C27	178.37 (11)
C7—C2—C3—C4	0.19 (18)	C22—N4—C24—O5	179.72 (11)
C1—C2—C3—C4	−178.61 (12)	C23—N4—C24—O5	2.12 (18)
C2—C3—C4—C5	0.21 (19)	C22—N4—C24—N5	−1.58 (16)
C3—C4—C5—C6	−0.25 (19)	C23—N4—C24—N5	−179.18 (10)
C4—C5—C6—C7	−0.11 (18)	C26—N5—C24—O5	−177.48 (11)
C5—C6—C7—C2	0.51 (18)	C25—N5—C24—O5	−1.59 (17)
C5—C6—C7—C8	179.56 (12)	C26—N5—C24—N4	3.83 (18)
C3—C2—C7—C6	−0.57 (18)	C25—N5—C24—N4	179.72 (10)
C1—C2—C7—C6	178.46 (11)	C24—N5—C26—O6	175.97 (11)
C3—C2—C7—C8	−179.80 (11)	C25—N5—C26—O6	0.09 (17)
C1—C2—C7—C8	−0.77 (13)	C24—N5—C26—C27	−4.66 (16)
C1—N1—C8—O2	−179.50 (11)	C25—N5—C26—C27	179.45 (10)
C9—N1—C8—O2	−1.95 (18)	N3—C22—C27—N2	−0.15 (13)
C1—N1—C8—C7	−0.18 (13)	N4—C22—C27—N2	179.47 (10)
C9—N1—C8—C7	177.36 (10)	N3—C22—C27—C26	178.33 (10)
C6—C7—C8—O2	0.7 (2)	N4—C22—C27—C26	−2.04 (18)
C2—C7—C8—O2	179.86 (12)	C21—N2—C27—C22	−0.09 (12)
C6—C7—C8—N1	−178.55 (12)	C20—N2—C27—C22	−179.68 (11)
C2—C7—C8—N1	0.60 (13)	C21—N2—C27—C26	−178.38 (12)
C8—N1—C9—C10	103.65 (13)	C20—N2—C27—C26	2.0 (2)
C1—N1—C9—C10	−79.09 (14)	O6—C26—C27—C22	−177.07 (11)
N1—C9—C10—C11	−173.66 (10)	N5—C26—C27—C22	3.61 (16)
C9—C10—C11—O3	−112.03 (13)	O6—C26—C27—N2	1.0 (2)
C9—C10—C11—O4	66.73 (13)	N5—C26—C27—N2	−178.35 (11)
C22—N3—C21—N2	−0.40 (13)		

### Hydrogen-bond geometry (Å, º)

**Table:**

*D*—H···*A*	*D*—H	H···*A*	*D*···*A*	*D*—H···*A*
O4—H4···N3^i^	0.84	1.83	2.6672 (13)	175
C3—H3*A*···O5^ii^	0.95	2.26	3.1447 (16)	155
C6—H6*A*···O3^iii^	0.95	2.31	3.2283 (16)	162
C20—H20*B*···O6^iv^	0.98	2.35	3.2559 (16)	154
C25—H25*A*···O6	0.98	2.28	2.7244 (18)	107
C25—H25*D*···O5	0.98	2.26	2.7152 (19)	107

Symmetry codes: (i) −*x*+1, −*y*, −*z*+2; (ii) −*x*, −*y*+1, −*z*+1; (iii) *x*, *y*+1, *z*; (iv) −*x*, −*y*+1, −*z*+2.
